# Validation of the PADIT (prevention of arrhythmia device infection trial) risk score for infection and infection subtypes

**DOI:** 10.1093/europace/euag016

**Published:** 2026-01-29

**Authors:** Mehrdad Golian, Zhe Li, Nicolas M Berbenetz, Roupen Odabashian, Mouhannad M Sadek, Vicente Corrales-Medina, Alper Aydin, Darryl R Davis, Martin S Green, Andres Klein, Girish M Nair, Pablo B Nery, F Daniel Ramirez, Calum Redpath, Simon P Hansom, Jodi D Edwards, Andrew D Krahn, David H Birnie

**Affiliations:** Division of Cardiology, Department of Medicine, University of Ottawa and University of Ottawa Heart Institute, H-1285D, 40 Ruskin St. Ottawa, Ottawa Ontario K1Y 4W7, Canada; Division of Cardiology, Department of Medicine, University of Ottawa and University of Ottawa Heart Institute, H-1285D, 40 Ruskin St. Ottawa, Ottawa Ontario K1Y 4W7, Canada; Division of Cardiology, Department of Medicine, University of Ottawa and University of Ottawa Heart Institute, H-1285D, 40 Ruskin St. Ottawa, Ottawa Ontario K1Y 4W7, Canada; Division of Cardiology, Department of Medicine, University of Ottawa and University of Ottawa Heart Institute, H-1285D, 40 Ruskin St. Ottawa, Ottawa Ontario K1Y 4W7, Canada; Division of Cardiology, Department of Medicine, University of Ottawa and University of Ottawa Heart Institute, H-1285D, 40 Ruskin St. Ottawa, Ottawa Ontario K1Y 4W7, Canada; Division of Infectious Diseases, Department of Medicine, University of Ottawa and the Ottawa Hospital, Ottawa, Ontario, Canada; Division of Cardiology, Department of Medicine, University of Ottawa and University of Ottawa Heart Institute, H-1285D, 40 Ruskin St. Ottawa, Ottawa Ontario K1Y 4W7, Canada; Division of Cardiology, Department of Medicine, University of Ottawa and University of Ottawa Heart Institute, H-1285D, 40 Ruskin St. Ottawa, Ottawa Ontario K1Y 4W7, Canada; Division of Cardiology, Department of Medicine, University of Ottawa and University of Ottawa Heart Institute, H-1285D, 40 Ruskin St. Ottawa, Ottawa Ontario K1Y 4W7, Canada; Division of Cardiology, Department of Medicine, University of Ottawa and University of Ottawa Heart Institute, H-1285D, 40 Ruskin St. Ottawa, Ottawa Ontario K1Y 4W7, Canada; Division of Cardiology, Department of Medicine, University of Ottawa and University of Ottawa Heart Institute, H-1285D, 40 Ruskin St. Ottawa, Ottawa Ontario K1Y 4W7, Canada; Division of Cardiology, Department of Medicine, University of Ottawa and University of Ottawa Heart Institute, H-1285D, 40 Ruskin St. Ottawa, Ottawa Ontario K1Y 4W7, Canada; Division of Cardiology, Department of Medicine, University of Ottawa and University of Ottawa Heart Institute, H-1285D, 40 Ruskin St. Ottawa, Ottawa Ontario K1Y 4W7, Canada; Division of Cardiology, Department of Medicine, University of Ottawa and University of Ottawa Heart Institute, H-1285D, 40 Ruskin St. Ottawa, Ottawa Ontario K1Y 4W7, Canada; Division of Cardiology, Department of Medicine, University of Ottawa and University of Ottawa Heart Institute, H-1285D, 40 Ruskin St. Ottawa, Ottawa Ontario K1Y 4W7, Canada; Division of Cardiology, Department of Medicine, University of Ottawa and University of Ottawa Heart Institute, H-1285D, 40 Ruskin St. Ottawa, Ottawa Ontario K1Y 4W7, Canada; Division of Cardiology, Department of Medicine, University of British Columbia, Vancouver, BC, Canada; Division of Cardiology, Department of Medicine, University of Ottawa and University of Ottawa Heart Institute, H-1285D, 40 Ruskin St. Ottawa, Ottawa Ontario K1Y 4W7, Canada

**Keywords:** Infection, Antibioticsbull Pacemaker, Implantable cardioverter defibrillator, Cardiac implantable electronic device, Pulse generator change, Device upgrade, Complication, Risk score, PADIT

## Abstract

**Aims:**

Cardiac implantable electronic device (CIED) infection carries a substantial burden of morbidity, mortality, and cost. The Prevention of Arrhythmia Device Infection Trial (PADIT) risk score improves identification of high-risk patients and may guide targeted strategies to reduce infection. Recent work has categorized CIED infection into localized pocket vs. systemic infection, with early reports suggesting different risk factors for each. However, no current risk score has been validated for infection subtypes.

Objectives

- Independently validate the PADIT infection risk score.
- Compare risk factors for infection subtypes.
- Assess PADIT performance in predicting subtype-specific infection.

**Methods and results:**

A prospective registry was initiated at the University of Ottawa Heart Institute in 2007 to capture all CIED procedures and prospectively identify infections in collaboration with the infection prevention team. PADIT risk score components were documented for each procedure. All suspected infections were adjudicated independently by two physicians (with a third if required), blinded to PADIT score and baseline variables, and subclassified as pocket or systemic infection. Logistic regression models were generated to validate PADIT performance for each subtype, with evaluation using Akaike and Bayesian information criteria (AIC/BIC), C-statistics, and calibration slope. Between 2007 and 2020, 14,225 procedures were performed (mean age 72 ± 14 years, 35% female, 70% new implants, 18% generator changes, 11% upgrades). A total of 103 infections (0.73%) were adjudicated, of which 71 (69%) were pocket and 32 (31%) systemic. The PADIT score showed good predictive performance with a C-statistic of 0.687 (95% CI 0.655–0.743), similar to the derivation cohort (0.702, 95% CI 0.661–0.741). Notably, the number of prior procedures was strongly associated with pocket infection but not systemic infection. PADIT discrimination was consistent across subtypes: pocket infection C-statistic 0.691 (95% CI 0.649–0.761) and systemic infection 0.746 (95% CI 0.707–0.848). Calibration slopes demonstrated good agreement between predicted and observed events, with the best fit for systemic infection.

**Conclusion:**

The PADIT score was independently validated with discrimination and calibration similar to the original derivation cohort. Importantly, prior procedures predicted pocket but not systemic infection. Overall, PADIT performed well in predicting both subtypes, with the strongest model fit observed for systemic infection.

## Graphical Abstract

**Graphical Abstract:**
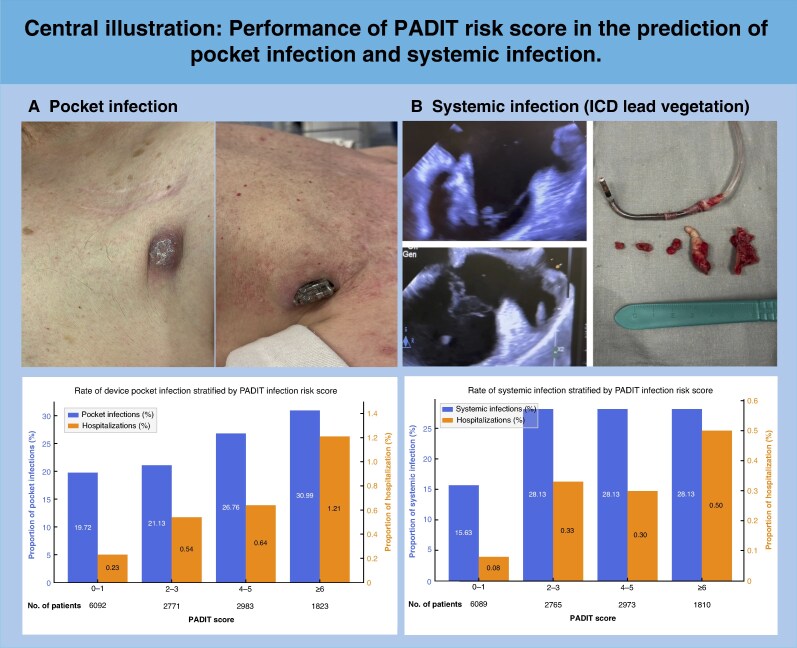

What’s new?

- The PADIT score was validated using a prospective observational cohort of 14 225 with a moderate C-statistic of 0.687 (95% CI: 0.655–0.743). This risk score has a C-statistic of 0.691 (95% CI: 0.649–0.761) for pocket infection and 0.746 (95% CI: 0.707–0.848) for systemic infection.
- The study findings suggest that the PADIT score demonstrates good predictive performance in identifying patients at risk for CIED infections, which supports its validity for clinical use. Moreover, the score effectively stratifies risk for both systemic and pocket infections, providing clinicians with a practical and comprehensive tool for infection risk assessment.

## Introduction

Cardiac implantable electronic device (CIED) infection carries a substantial risk of morbidity and mortality.^1,2^ Preventative approaches, such as perioperative intensified antibiotic therapy and the use of antibiotic impregnated pouches, have demonstrated benefits in reducing CIED infections.^3,4^ However these interventions are costly; therefore leveraging validated risk scores to identify patients at the highest risk can lead to cost savings.^5^ Estimating the risk of infection from complex procedures is also critical for shared decision making around procedure options.

Several perioperative CIED infection risk prediction scores have previously been developed.^2,6–11^ While some used only pre-operative variables, others include perioperative factors. The largest study to date used data from 19 603 patients in the randomized PADIT trial and identified five independent pre-operative predictors of device infection to develop the PADIT risk scoring system.^2^ Although the PADIT risk score has been independently validated in several other studies; there are some issues with the validation process in all of these studies.^6,11,12^ The PADIT score was designed to be a pre-implant risk stratification tool and thus relies exclusively on pre-procedural variables. A pre-implant risk score helps with shared decision-making, and targeted preventative strategies, in contrast to scores incorporating perioperative variables that are not available at the time of initial clinical decision-making. Also very importantly, recent work has begun to divide CIED infection into two subtypes, either as localized pocket CIED infection or systemic CIED infection, with or without lead-associated endocarditis.^13^ This distinction is clinically important, as patients with systemic infection have substantially worse outcomes, including higher mortality, morbidity, and healthcare utilization.^2,5,14,15^ Initial reports suggest potential differences in risk factors for each subtype, but additional data is needed.^13^ Further, none of the existing infection risk scores have been validated for infection subtype.

Our study had three aims:

- Independently validate the PADIT infection risk score.
- Compare risk factors for subtypes of infection
- Explore the efficacy of the PADIT score at predicting the subtypes of device infection.

## Methods

### Patients and registry details

A prospective registry of all individuals who underwent a CIED implant procedure was started at the University of Ottawa Heart Institute (UOHI) in January 2007. In the Canadian single -payer national health service, care is regionalized to various extents. In our region of 1.5 million people all CIED implants and follow-up for all patients are performed at our centre. Because our institution provides both comprehensive device clinic follow-up and a lead extraction service, all patients undergoing device implantation complete their follow-up at our centre. Also we are the only device extraction centre for our population.^16^ The registry was initiated in collaboration with the Division of Infectious Diseases to prospectively identify all individuals with CIED infections. The current project was reviewed by our institutional ethics committee who indicated that the project falls within the context of quality initiative and quality improvement, and hence, as per the Tri-Council Policy Statement 2, Article 2.5, full review by the ethics committee was not required.

### Routine peri-operative infection prevention measures

During the study period, UOHI institutional protocol mandated that surgical site preparation included hair removal, a 2% chlorhexidine skin wash, followed in 2 min by a 4% alcohol skin wash and cefazolin within 1 h prior to the procedure. In cases of penicillin allergy, vancomycin was used. All patients routinely had the device pocket washed with bacitracin prior to closure of the incision. The only exception to this standardized regime was during the period that our centre participated in the Prevention of Arrhythmia Device Infection Trial (PADIT) study between 2012 and 2015. PADIT was a cluster randomized four period crossover study comparing incremental (pre-procedural cefazolin plus vancomycin, intraprocedural bacitracin pocket wash, and 2-day post-procedural oral cephalexin) to conventional (pre-procedural cefazolin only) perioperative CIED management.^17^ Our site underwent four sequences of protocol change every 6 months as follows: incremental, conventional, incremental, and conventional.^4,17^ In addition, our center contributed to the World-Wide Randomised Antibiotic Envelope Infection Prevention (WRAPIT) study (2012–2016) that investigated the effectiveness of using an antibiotic-impregnated pouch (Tyrx) during high-risk device surgery, as compared to standard of care. As part of this study, we used a total of thirty-seven Tyrx pouches.^3^

### Outcome

For the present validation study, the primary outcome was hospitalisation for device infection. This was further subclassified as pocket infection and/or systemic infection (defined as bloodstream infection and/or endocarditis). If a patient had both a pocket infection and systemic infection, then for the purposes of this study they were counted as systemic infection. The definitions were exactly as used in the PADIT study.^4^ In brief, local pocket infection was characterized by mechanical erosion of the CIED device or the presence of erythema and purulent discharge at the generator-pocket site without positive blood culture or lead vegetation, necessitating device extraction. Systemic CIED infection was defined as systemic symptoms arising from a bloodstream infection, with or without accompanying pocket infection, also requiring device extraction. The adjudication process also followed the exact protocol of the PADIT study. Blinded adjudication of all potential endpoints was performed by two investigators (NB and RO) blinded to treatment received, with all discrepancies resolved by an additional investigator (MG).

**Variable Definitions (** *Variables used and defined as in PADIT with exception of immunocompromised):* ^4^

- Renal insufficiency in this analysis was uniformly defined as estimated glomerular filtration rate < 30 mL/min, as done in PAIDT. PADIT-specified threshold was retained to ensure methodological fidelity with the derivation cohort.
- Procedure type definitions:
  1. Pacemaker: new pacemaker or pacemaker generator change
  2. Implantable cardioverter defibrillator (ICD): new ICD or ICD generator change
  3. Cardiac resynchronization therapy (CRT): new CRT pacemaker or defibrillator or CRT generator change
  4. Revision/upgrade: Pocket and/or lead revision and/or system upgrade [i.e. addition of new lead(s)]
    It is important to note that the four groups within procedure type are mutually exclusive. For example, if a patient is undergoing upgrade to CRT, then they are counted as ‘revision/upgrade’.
    Also, leadless devices were excluded, and hematoma evacuation procedures were classified as revisions.
- Immunocompromised:

In contrast in the derivation cohort immunocompromised was defined as: receiving therapy that suppresses resistance to infection (e.g. immunosuppression, chemotherapy, radiation, long-term or recent high-dose steroids) or having a disease that is sufficiently advanced to suppress resistance to infection (e.g. leukaemia, lymphoma, HIV infection).^2^

### PADIT score calculation

CIED infection risk using the PADIT score was determined by identifying five variables: prior procedures (P), age (A), renal insufficiency (D), immunocompromised status (I), and procedure type (T). This yields a score that ranges from 0 to 15 points. The score stratifies patients into three risk categories: low risk (0 to 4 points), intermediate risk (5 to 6 points), and high risk (≥7 points). These risk categories correspond to distinct rates of hospitalisation for infection, namely 0.51% for low-risk patients, 1.42% for intermediate-risk patients, and 3.41% for high-risk patients.^2^

### Statistical analysis and ethics

Continuous variables and categorical variables were summarized as mean ± standard deviation (SD) and frequency (percentage), respectively. Baseline characteristics, including age, sex, procedure type (pacemaker, implantable cardioverter defibrillator, cardiac resynchronisation therapy, lead revision), number of previous procedures, renal insufficiency, and immunocompromised status, were compared between patients with and without hospitalisation for device infection, using two-sample t-tests for continuous variables and a Chi-square or Fisher’s exact test for categorical variables, as appropriate. The PADIT risk score was calculated using the predefined scoring system, as described above. To validate the risk score, logistic regression models were built to estimate the predictive utility of the PADIT score for the outcome of hospitalization for device infection and infection subtype. To mitigate the potential for model overfitting, Lasso logistic regression was used to improve model prediction accuracy. Odds ratios, ß coefficients, and *P*-values were reported to estimate statistical associations between each predictor and the outcome. A *P*-value less than 0.05 was considered statistically significant.

Performance metrics, including Akaike information criterion (AIC), Bayesian information criterion (BIC), c-statistics, and calibration slope, were reported to evaluate the performance of the PADIT score for the prediction of infection subtypes.^18,19^ The AIC and BIC assess model fit, with a lower value indicating a better model fit with the observed data. Discrimination was evaluated using the C-statistic, which calculates the probability that individuals who had the outcome have a higher PADIT risk than those who did not have the outcome. Calibration assesses the degree of agreement between observed and predicted probabilities, as reported using the calibration slope. Sensitivity analyses were conducted to test whether model performance results remained robust after removing the variable indicating immunocompromised status.

## Results

### Participant characteristics

A total of 14 225 individuals who underwent a CEID procedures were included in the registry between 2007 and 2020. There were 103 (0.73%) adjudicated primary endpoints (hospitalization for device infection). Of these, 71/103 (69%) were pocket infection alone and 32/103 (31%) systemic infection. *Table 1* (note this table has been previously published)^20^ shows the comparison of baseline characteristics in patients with and without CIED infection. Patients with CIED infection were significantly younger than patients without infection. Compared with patients without infection, a substantially higher proportion of patients with CIED had undergone more than two procedures previously.

**Table 1:** Baseline characteristics in patients with and without adjudicated infection

	Adjudicated infection (*n* = 103)	No adjudicated infection (*n* = 14 122)	*P* value
**Age, years**	66.0 ± 15.0	72.2 ± 13.8	<0.01
**Female**	27 (26.2%)	4891 (34.6%)	0.09
**Procedure type**			
**Pacemaker**	32 (31.1%)	7784 (55.1%)	<0.01
**Implantable cardioverter defibrillator**	33 (32.0%)	3259 (23.1%)	0.04
**Cardiac resynchronisation therapy**	20 (19.4%)	1462 (10.4%)	<0.01
**Lead revision**	18 (17.5%)	1617 (11.5%)	0.08
**Renal insufficiency**	7 (6.8%)	888 (6.3%)	0.99
**Immunocompromised**	3 (3.0%)	438 (3.1%)	1.00
**Number of previous procedures**			
**1**	63 (61.2%)	9942 (70.4%)	0.05
**2**	18 (17.5%)	2939 (20.8%)	0.47
**>2**	22 (21.4%)	1241 (8.8%)	<0.01
**PADIT Score**	3.99 ± 2.46	2.54 ± 2.43	<0.01

### Results of logistic regression for the predicting CIED infection

*Table 2* reports the odds ratios and ß coefficient of each predictor and CIED infection in comparison with the results obtained from the derivation cohort. Similar to the results from the derivation cohort, age <60, implantable cardioverter defibrillator, cardiac resynchronisation, and the number of procedures >2 were significantly associated with CIED infection. Renal insufficiency and immunocompromised status were significant predictors in the derivation cohort but not in this validation cohort. PADIT score modelling using the current cohort is shown in *Table 2* [C- statistic = 0.687 (95% CI: 0.655–0.743)]. The original modelling from the derivation cohort is shown in comparison (C- statistic = [0.702 (95% CI: 0.661–0.741)].

**Table 2:** Validation of PADIT score in the cohort with and without adjudicated infection with comparison to the derivation cohort^2^

	Data from the validation cohort			Data from the published derivation cohort		
	Odds ratio (95% CI)	ß coefficient	*P* value	Odds ratio (95% CI)	ß coefficient	*P* value
**Age [reference =** **>** **70 years old]**						
**60–69**	1.74 (1.06–2.84)	0.5583	0.025			
**<60**	2.09 (1.26–3.43)	0.7390	0.003		−0.0274	0.018
**Procedure Type [reference** **=** **pacemaker]**						
**Implantable cardioverter defibrillator**	1.84 (1.09–3.13)	0.6135	0.022	1.77 (1.09–2.87)	0.5717	0.020
**Cardiac resynchronisation therapy**	2.59 (1.43–4.60)	0.9524	0.001	2.73 (1.72–4.31)	1.0026	<0.001
**Lead revision**	1.97 (0.97–4.02)	0.6776	0.061	4.01 (2.62–6.13)	1.3881	<0.001
**Renal insufficiency**	1.23 (0.52–2.51)	0.2138	0.589	1.45 (1.00–2.09)	0.3697	0.047
**Immunocompromised** ^a^	0.95 (0.23–2.54)	−0.0554	0.925	2.28 (1.05–4.96)	0.8261	0.037
**Number of procedures [reference =1]**						
**2**	0.82 (0.43–1.47)	−0.1993	0.522	1.51 (0.99–2.32)	0.4146	0.058
**>2**	2.23 (1.23–3.94)	0.8015	0.005	3.43 (2.14–5.54)	1.2321	<0.001
**Intercept**	—	−5.7052	<0.001	—	−3.3207	0.001

^a^Immunocompromised was defined as receiving therapy that suppresses resistance to infection (e.g. immunosuppression, chemotherapy, radiation, long-term or recent high-dose steroids) or having a disease that is sufficiently advanced to suppress resistance to infection (e.g. leukemia, lymphoma, HIV infection)

### Predictors of subtypes of infection

Among the 103 patients who had CIED infection, 71(69%) had adjudicated pocket infections, and 32 (31%) had systemic infection endpoints. *Table 3* shows the characteristics of patients stratified by infection subtype. Compared with patients without a CIED infection (2.54 ± 2.43), patients with pocket (4.01 ± 2.43) or systemic infection (3.94 ± 2.54) had significantly higher PADIT scores.

**Table 3:** Characteristics of patients stratified by infection subtype compared to the non-infected cohort. (*P* values are for comparisons between individual subtypes and no infection)

	No pocket infection (*n* = 14 122)	Pocket infection (*n* = 71)	*P* value	Systemic infection (*n* = 32)	*P* value
**Age, years**	72.2 ± 13.8	67.0 ± 15.0	<0.01	63.8 ± 14.9	<0.01
**Female**	4891 (34.6%)	21 (29.6%)	0.44	6 (18.8%)	<0.01
**Procedure type**					
**Pacemaker**	7784 (55.1%)	24 (33.8%)	<0.01	8 (25%)	<0.01
**Implantable cardioverter defibrillator**	3259 (23.1%)	21 (29.6%)	0.24	12 (37.5%)	0.28
**Cardiac resynchronisation therapy**	1462 (10.4%)	14 (19.7%)	0.01	6 (18.8%)	0.74
**Lead revision**	1617 (11.5%)	12 (16.9%)	0.21	6 (18.8%)	0.54
**Renal insufficiency**	888 (6.3%)	6 (8.5%)	0.61	1 (3.1%)	0.14
**Immunocompromised**	438 (3.1%)	1 (1.4%)	0.63	2 (6.3%)	1.00
**Number of previous procedures**					
**1**	9942 (70.4%)	38 (53.5%)	<0.01	25 (78.1%)	<0.01
**2**	2939 (20.8%)	15 (21.1%)	1.00	3 (9.4%)	<0.01
**>2**	1241 (8.8%)	18 (25.4%)	<0.01	4 (12.5%)	0.46
**PADIT SCORE**	2.54 ± 2.43	4.01 ± 2.43	<0.01	3.94 ± 2.54	<0.01

*Table 4* presents the associations between predictors and pocket infection. Age 60–69 (OR, 1.85; 95% CI, 1.03–3.28), cardiac resynchronisation therapy (OR, 2.24; 95% CI, 1.10–4.40) and number of previous procedures > 2 (OR, 3.60; 95% CI, 1.88–6.62) were significantly associated with pocket infection. *Table 5* shows the results of logistic regression for systemic infection, and age <60 (OR, 2.51; 95% CI, 1.05–6.04), cardiac resynchronisation therapy (OR, 3.60; 95% CI, 1.15–10.65) and lead revision (OR, 18.01; 95% CI, 2.70–36.19) were statistically significant predictors for systemic infection. In contrast to the findings for pocket infection, the number of previous procedures was not associated with a greater risk of systemic infection.

**Table 4:** Full prediction model for pocket infection (logistic regression)

	Primary analysis		
	Odds ratio (95% CI)	ß coefficient	*P* value
**Age [reference category =** **>** **70 years old]**			
**60–69**	1.85 (1.03–3.28)	0.6176	0.035
**<60**	1.87 (0.99–3.43)	0.6265	0.046
**Procedure Type [reference category** **=** **pacemaker]**			
**Implantable cardioverter defibrillator**	1.61 (0.86–3.01)	0.4762	0.135
**Cardiac resynchronisation therapy**	2.24 (1.10–4.40)	0.8055	0.021
**Lead revision**	1.25 (0.55–2.80)	0.2262	0.582
**Renal insufficiency**	1.56 (0.60–3.36)	0.4442	0.304
Immunocompromised**	0.43 (0.02–1.97)	−0.8394	0.405
**Number of procedures [reference category =1]**			
**2**	1.35 (0.67–2.56)	0.2982	0.381
**>2**	3.60 (1.88–6.62)	1.2813	<0.001
**Intercept**	—	−6.1291	<0.001

*For pocket infections, the primary model had an AIC of 863.5, BIC of 938.7, and c-statistic of 0.691 (0.649–0.761), with calibration of 1.000 (0.675–1.320); sensitivity analysis showed AIC 930.1, BIC 862.4, c-statistic 0.693 (0.642–0.760), and calibration 1.000 (0.672–1.323).

** Immunocompromised was defined as receiving therapy that suppresses resistance to infection (e.g. immunosuppression, chemotherapy, radiation, long-term or recent high-dose steroids) or having a disease that is sufficiently advanced to suppress resistance to infection (e.g. leukemia, lymphoma, HIV infection)

**Table 5:** Full prediction model for systemic infection (logistic regression)

	Primary analysis		
	Odds ratio (95% CI)	ß coefficient	*P* value
**Age [reference =** **>** **70 years old]**			
**60–69**	1.53 (0.58–3.80)	0.4222	0.372
**<60**	2.51 (1.05–6.04)	0.9205	0.037
**Procedure Type [reference** category **=** **pacemaker]**			
**Implantable cardioverter defibrillator**	2.50 (0.96–6.83)	0.9159	0.064
**Cardiac resynchronisation therapy**	3.60 (1.15–10.65)	1.2802	0.021
**Lead revision**	18.01 (2.70–36.19)	2.8914	0.011
**Renal insufficiency**	0.56 (0.03–2.66)	−0.5766	0.572
Immunocompromised**	2.35 (0.38–7.91)	0.8529	0.247
**Number of procedures [reference category =1]**			
**2**	0.08 (0.004–0.04)	−2.4927	0.027
**>2**	0.25 (0.01–1.34)	−1.3678	0.199
**Intercept**	—	−6.8367	<0.001

* Systemic infection modelling showed the strongest discrimination, with an AIC of 444.5, BIC of 519.7, c-statistic of 0.746 (0.707–0.848), and calibration of 1.000 (0.599–1.431). The sensitivity analysis for this group yielded AIC 443.5, BIC 511.2, c-statistic 0.741 (0.697–0.831), and calibration 1.000 (0.589–1.446)

** Immunocompromised was defined as receiving therapy that suppresses resistance to infection (e.g. immunosuppression, chemotherapy, radiation, long-term or recent high-dose steroids) or having a disease that is sufficiently advanced to suppress resistance to infection (e.g. leukemia, lymphoma, HIV infection)

### Performance of the PADIT score in predicting infection subtype

*Table 6* shows the performance of PADIT risk score in the prediction of infection and the subtypes of infection. When predicting adjudicated infection using data from the whole cohort, the PADIT score showed a C-statistic of 0.687 (95% CI, 0.644–0.743). When predicting different subtypes of infection, the score showed a C-statistic of 0.691 (95% CI: 0.649–0.761) for pocket infections, and 0.746 (95% CI: 0.707–0.848) for systemic infections. The slope of calibration was 1.000 for all types of infections and indicated the predicted probability of having the outcome matches the observed data well. In terms of model fit, the PADIT score achieved the best fit when predicting systemic infection using the cohort data.

**Table 6:** Performance of PADIT risk score in the prediction of adjudicated infection, pocket infection, and systemic infection

	AIC	BIC	c-statistic	Calibration
**Adjudicated infection**	1178.6	1253.9	0.687 (0.655–0.743)	1.000 (0.707–1.294)
**Sensitivity analysis**	1176.6	1244.3	0.687 (0.646–0.746)	1.000 (0.707–1.296)
**Pocket infection**	863.5	938.7	0.691 (0.649–0.761)	1.000 (0.675–1.320)
**Sensitivity analysis**	930.1	862.4	0.693 (0.642–0.760)	1.000 (0.672–1.323)
**Systemic infection**	444.5	519.7	0.746 (0.707–0.848)	1.000 (0.599–1.431)
**Sensitivity analysis**	443.5	511.2	0.741 (0.697–0.831)	1.000 (0.589–1.446)

Sensitivity analyses were conducted to test whether model performance results remained similar after removing the variable immunocompromised status.

Due to the different measures used to define immunocompromised status, we performed a sensitivity analysis; as shown in *Table 6*, removing this variable did not significantly change the model results.

## Discussion

Our study had three main findings. Firstly, the PADIT risk score assessed using our regional CIED centre cohort had very similar results to the original derivation cohort (C statistic of 0.687 (95% CI: 0.655–0.743) compared to a C statistic of 0.702 (95% CI: 0.661–0.741).^2^ Secondly, the present study presents novel data evaluating the risk factors for subtypes of device infection and found a major difference among subtypes with a significant association between the number of prior procedures and pocket infection, but no association between prior procedures and increased risk of systemic infection. Thirdly, we assessed the performance of the score for the subtypes of infection and found it performed well for patients with pocket infection and slightly better for systemic infection.

The present study confirms the moderate accuracy of the PADIT score for predicting the risk of subsequent device infection. In evaluating the components of the PADIT score in our validation cohort, the presence of renal insufficiency (OR 1.23 (0.52–2.51, *P* = 0.59) was not independently associated with higher infection risk. However, this trend was similar to the derivation cohort, which showed renal insufficiency had an OR 1.45 (1.00–2.09, *P* = 0.047).^2^ It is also noteworthy to acknowledge that the weakest correlate with infection is renal insufficiency; also, that other infection studies and risk scores have used alternative eGFR thresholds, and that the method used for eGFR calculation has not been uniformly specified, which may contribute to heterogeneity in reported associations with infection.^2,21–23^

Although the PADIT infection risk score has previously been assessed in several studies, our analysis is the first to precisely reproduce the same methodology (definition and event adjudication) as used in the derivation cohort. Ahmed et al. conducted a retrospective validation using a U.S. health claims database of 51 623 patients, reporting a 28% relative risk increase in CIED-related infection for every 1-point increase in the PADIT score (c-statistic: 0.76). Boriani et al. evaluated the predictive performance of in 2675 patients with 28 documented infections and found a C-statistic of 0.64. Similarly, Heide et al. validated the PADIT score in a retrospective study of 2333 patients, reporting a C-statistic of 0.7.^12^ Maclean et al. compared the predictive accuracy of the PADIT and BLISTER (Blood results, long procedure time, Immunosuppressed, Sixty years old or younger, Type of procedure, Early re-intervention, Repeat procedure) scores in a cohort of 7383 patients with 59 infections.^11^ The BLISTER score achieved a higher C-statistic of 0.78 (95% CI: 0.71–0.85) compared to the PADIT score.^11^ However, it is important to note that the BLISTER score incorporates perioperative variables and hence is not directly comparable.

Recent work has begun to divide CIED infection into two subtypes, either localized pocket CIED infection or systemic CIED infection with or without lead-associated endocarditis.^13^ CIED infection subtype carries important prognostic information. Massaro et al. demonstrated that systemic infection (without pocket infection) is associated with significantly worse outcomes compared with pocket infection, whether occurring in isolation or with systemic involvement.^15^ Similarly, in a retrospective analysis of 3847 patients, Jacheć et al. demonstrated significantly higher three-year mortality among patients with systemic infection-related lead endocarditis compared with those presenting with isolated pocket infection (55.09% vs. 27.75%; *P* < 0.001).^24^ Initial data suggest some differences in risk factors for each subtype.^13^ In our study, independent risk factors for pocket infection were younger age, the type of procedure and the number of prior procedures, with a history of more than two implant site procedures being the strongest predictor. For systemic infection, younger age, male sex and procedure type were all independently associated with increased risk, whereas the number of prior procedures was not associated with increased risk of systemic infection. Indeed, there was a negative association between two or more procedures and systemic infection. This finding is consistent with prior observations from a substudy of the European Lead Extraction Controlled (ELECTRa) registry, which included 1850 patients undergoing lead extraction for infection. In that analysis, repeat procedures and device upgrades were not associated with an increased risk of systemic infection, with comparable proportions observed for pocket vs. systemic infection (17.8% vs. 21.0%; *P* = 0.0855 and 14.96% vs. 12.79%; *P* = 0.1982, respectively*)*.^14^ While previous studies have reported the strongest risk factor for pocket infection was a higher number of procedures (OR of 3.6 (95% CI = 1.88–6.62) and the strongest risk factor for systemic infection was lead revision (OR 18.01 (95% C = 2.70–36.19), the mechanisms of these findings are not clear. One hypothesis relates to the differing postulated pathophysiology. Specifically, in pocket infections, bacteria are introduced from the skin at the time of the procedure, and the immune system response determines whether an infection occurs. Furthermore, the immune response is more likely to be successful in a relatively vascular setting (i.e. *de novo* procedure) than subsequent procedure (with an established avascular capsule). In contrast, systemic infection is either due to bacteria introduced from the skin directly onto the leads or seeding to the leads during another systemic infection.

### Limitations

The primary limitation of our study is the potential for missing predictors. One particular example is that we did not collect information regarding oral anticoagulation (OAC) and antiplatelet therapy. Prior studies indicate that pocket hematoma is an important predictor of infection, and perioperative management of OACs and antiplatelets can affect the frequency of hematoma formation (and in turn the risk of infection).^16,25,26^ However, with major advances in the management of these agents, the rates of clinically significant hematoma are vastly reduced and thus much less relevant.^16,27^ A second limitation is that we had to define immunocompromised differently from the validation cohort. However, a sensitivity analysis showed the score performed similarly with and without this variable. We acknowledge that the limited number of infection events in our cohort precluded further subdivision of infection phenotypes, including systemic infection without pocket involvement vs. pocket infection with systemic involvement. This remains an important area for future investigation.

### Conclusions and clinical implications

Using identical methodology (variable definitions and adjudication process) to that used in the derivation cohort, we confirmed that the PADIT infection risk score performed moderately well. Secondly, we explored risk factors for subtypes of device infection and found one major difference: specifically, there was no association between the number of prior procedures and increased risk of systemic infection. Thirdly, we assessed the performance of the score for the subtypes of infection and found it performed well for patients with pocket infection and slightly better for systemic infection.

## Data Availability

The data underlying this study cannot be shared publicly due to ethical and privacy restrictions related to participant confidentiality and institutional review board requirements. De-identified data may be made available from the corresponding author upon reasonable request, subject to institutional approval.
